# *Sensors* Best Paper Award 2012

**DOI:** 10.3390/s120101127

**Published:** 2012-01-23

**Authors:** Ophelia Han

**Affiliations:** MDPI AG, Postfach, CH-4005 Basel, Switzerland and MDPI Branch Office, Beijing 101101, China; E-Mail: ophelia.han@mdpi.com; Tel.: +86-10-5901-1009; Fax: +86-10-5901-1089

Since 2011, *Sensors* has been instituting an annual award to recognize outstanding papers that are related to sensing technologies and applications and meet the aims, scope and high standards of this journal [1]. To improve the timeliness of the award, we have decided that starting from next year, only papers published in the preceding three years will be eligible for the competition.

In this year, nominations were made by the Section Editor-in-Chiefs of *Sensors* from a pool of all papers published in *Sensors* from 2006 to 2008, in which reviews and articles were considered separately. We gladly announce that the following five papers have won the *Sensors* Best Paper Award in 2012.

## Article Award:

### 

#### 1^st^ Prize

**Hongying Zhu, Jonathan D. Suter, Ian M. White and Xudong Fan**

Aptamer Based Microsphere Biosensor for Thrombin Detection

*Sensors* **2006**, *6*(8), 785–795; doi:10.3390/s6080785

Available online: http://www.mdpi.com/1424-8220/6/8/785/

#### 2^nd^ Prize

**Veronika Supalkova, Dalibor Huska, Vaclav Diopan, Pavel Hanustiak, Ondrej Zitka, Karel Stejskal, Jiri Baloun, Jiri Pikula, Ladislav Havel, Josef Zehnalek, Vojtech Adam, Libuse Trnkova, Miroslava Beklova and Rene Kizek**

Electroanalysis of Plant Thiols

*Sensors* **2007**, *7*(6), 932–959; doi:10.3390/s7060932

Available online: http://www.mdpi.com/1424-8220/7/6/932/

#### 3^rd^ Prize

**Cédric Cochrane, VladanKoncar, Maryline Lewandowski and Claude Dufour**

Design and Development of a Flexible Strain Sensor for Textile Structures Based on a Conductive

Polymer Composite

*Sensors* **2007**, *7*(4), 473–492; doi:10.3390/s7040473

Available online: http://www.mdpi.com/1424-8220/7/4/473/

## Review Award:

### 

#### 1^st^ Prize

**Dorothee Grieshaber, Robert MacKenzie, Janos Vörös and Erik Reimhult**

Electrochemical Biosensors - Sensor Principles and Architectures

*Sensors* **2008**, *8*(3), 1400–1458; doi:10.3390/s8031400

Available online: http://www.mdpi.com/1424-8220/8/3/1400/

#### 2^nd^ Prize

**HuaBai and Gaoquan Shi**

Gas Sensors Based on Conducting Polymers

*Sensors* **2007**, *7*(3), 267–307; doi:10.3390/s7030267

Available online: http://www.mdpi.com/1424-8220/7/3/267/

The prize awarding committee merits the article “Aptamer Based Microsphere Biosensor for Thrombin Detection” as “a well-written paper reporting very interesting scientific work, which is the type of paper we want to encourage submission of at *Sensors*”, which reported “an interesting detection principle nicely coupled to the use of aptamers, a relative new type of biological reagent”. The article “Electroanalysis of Plant Thiols” reported “… a nice, important application of an electrochemical technique…”.

These five exceptional papers are valuable contributions to *Sensors* and the sensing field. On behalf of the Prize Awarding Committee and the Editorial Board of *Sensors*, we would like to congratulate these five teams for their excellent work. In recognition for their accomplishment, Drs. Xudong Fan, Rene Kizek and Vladan Koncar will receive a prize of 1000 CHF, 800 CHF, and 500 CHF, and Drs. Erik Reimhult and Gaoquan Shi will receive a prize of 800 CHF and 500 CHF, respectively, and the privilege to publish an additional paper free of charge in open access format in *Sensors*, after normal peer-review.

Prize Awarding Committee

*Editor-in-Chief*, Section ‘Physical Sensors’

**Prof. Dr. Craig A. Grimes**

State Key Laboratory of Materials-Oriented Chemical Engineering, Department of Chemical

Engineering, Nanjing University of Technology, Nanjing, China

E-Mail: craig.grimes40@gmail.com

*Editor-in-Chief*, Section ‘Remote Sensors’

**Dr. Assefa M. Melesse**

Department of Environmental Studies, ECS 339, Florida International University, 11200 SW 8th

Street, Miami, FL 33199, USA

Tel. +1-305-348-6518; Fax: +1-305-348-6137

Website: http://www.fiu.edu/~melessea/

E-Mail: assefa.melesse@fiu.edu

*Editor-in-Chief*, Section ‘Chemical Sensors’

**Prof. Dr. W. Rudolf Seitz**

Analytical Chemistry, Department of Chemistry, University of New Hampshire, Durham,

NH 03824-3598, USA

Tel. +1-603-862-2408; Fax: +1-603-862-4278

Website: http://www.unh.edu/chemistry/faculty/seitz_w.html

E-Mail: wrs@cisunix.unh.edu

*Editor-in-Chief*, Section ‘Biosensors’

**Dr. Alexander Star**

Department of Chemistry, University of Pittsburgh, 219 Parkman Avenue, Pittsburgh, PA 15260, USA

Tel. +1-412-624-6493; Fax: +1-412-624-4027

Website: http://www.pitt.edu/~astar/

E-Mail: astar@pitt.edu

*Editor-in-Chief*, Section ‘Sensor Networks’

**Dr. Tim Wark**

CSIRO ICT Centre, 1 Technology Ct, QCAT, Pullenvale, QLD 4069, Australia

Tel. +61-3327-4042; Fax: +61-3327-4455

Website: http://www.ict.csiro.au/staff/tim.wark/

E-Mail: tim.wark@csiro.au

Managing Editor

**Dr. Ophelia Han**

MDPI Beijing Office, Liyuanbeijie Road 186, Suite 307, Liyuan Town, Tongzhou District, Beijing

101101, China

E-Mail: ophelia.han@mdpi.com
